# The current and future distribution of the yellow fever mosquito (*Aedes aegypti*) on Madeira Island

**DOI:** 10.1371/journal.pntd.0010715

**Published:** 2022-09-12

**Authors:** José Maurício Santos, César Capinha, Jorge Rocha, Carla Alexandra Sousa

**Affiliations:** 1 Centre for Geographical Studies, Institute of Geography and Spatial Planning, University of Lisbon, Lisbon, Portugal; 2 Associated Laboratory TERRA, Lisbon, Portugal; 3 Global Health and Tropical Medicine, Instituto de Higiene e Medicina Tropical, Universidade Nova de Lisboa, Lisbon, Portugal; University of Hong Kong, HONG KONG

## Abstract

The *Aedes aegypti* mosquito is the main vector for several diseases of global importance, such as dengue and yellow fever. This species was first identified on Madeira Island in 2005, and between 2012 and 2013 was responsible for an outbreak of dengue that affected several thousand people. However, the potential distribution of the species on the island remains poorly investigated. Here we assess the suitability of current and future climatic conditions to the species on the island and complement this assessment with estimates of the suitability of land use and human settlement conditions. We used four modelling algorithms (boosted regression trees, generalized additive models, generalized linear models and random forest) and data on the distribution of the species worldwide and across the island. For both climatic and non-climatic factors, suitability estimates predicted the current distribution of the species with good accuracy (mean area under the Receiver Operating Characteristic curve = 0.88 ±0.06, mean true skill statistic = 0.72 ±0.1). Minimum temperature of coldest month was the most influential climatic predictor, while human population density, residential housing density and public spaces were the most influential predictors describing land use and human settlement conditions. Suitable areas under current climates are predicted to occur mainly in the warmer and densely inhabited coastal areas of the southern part of the island, where the species is already established. By mid-century (2041–2060), the extent of climatically suitable areas is expected to increase, mainly towards higher altitudes and in the eastern part of the island. Our work shows that ongoing efforts to monitor and prevent the spread of *Ae*. *aegypti* on Madeira Island will have to increasingly consider the effects of climate change.

## Author summary

The *Aedes aegypti* mosquito is an invasive species on Madeira Island and recently responsible for a dengue outbreak that affected more than 2000 people. To help control the activity of this mosquito, the local health authorities have an entomological surveillance program in place throughout the island. However, the full extent of the areas that can be colonized by this species remains unknown. We estimate the current and future potential distribution of *Ae*. *aegypti* on Madeira Island accounting for climatic, land use and human settlement conditions. Our results suggest that suitable conditions are predominantly distributed along the southern coast of the island. However, as climate change progresses, climatically suitable areas are expected to increase, particularly at mid-altitudes and in eastern part of the island. Minimum temperature of the coldest month was the most influential predictor variable in climatic suitability models, while human population density, housing density and public spaces were the most influential in models of land use and human settlement suitability. Our work provides valuable insight on the potential distribution of *Ae*. *aegypti* on Madeira Island, which can be used to inform ongoing and future monitoring and prevention initiatives.

## 1. Introduction

Increasing globalization in the movement of people and goods is enabling the dispersal of non-native species worldwide [1,2]. The yellow fever mosquito (*Ae*. *aegypti*) benefitted greatly from this process, currently invading many tropical and subtropical regions of the world [3]. These invasions are of major health concern, as the species is the main vector of arboviral diseases such as dengue, chikungunya, yellow fever and Zika [4,5].

*Ae*. *aegypti* was first identified on Madeira Island in 2005 [6]. Since then, it has widened its range significantly, colonizing several of the most densely populated areas on the southern edge of the island [6,7]. In 2012 the species caused a dengue outbreak, with 1080 confirmed infections and over 2000 probable cases [8]. To help reduce the risk of future outbreaks, local health authorities now closely monitor species distribution across the island [9]. However, the invasion process is still ongoing and the full extent of areas that could be colonized by the species remains unknown.

The potential distribution of *Ae*. *aegypti* is challenging to predict. The physiology of the species is strongly influenced by temperature and humidity [10,11], and climatic conditions are recognized as the main determinant of its distribution at coarse resolutions [11]. At finer scales, factors related to land use and human settlement can also play an important role [12]. Built environments in particular can supply thermal shelter and water-retaining conditions, sometimes allowing the species to establish locally, even if macroclimatic conditions are not favorable [13].

Madeira island has a varied and contrasting climatic geography owing to its location in the transition from subtropical to temperate latitudes, steep topography and longitudinally elongated shape. Thus, it is unclear whether *Ae*. *aegypti* will be able to endure the climatic conditions of many areas where it has not yet been recorded, particularly those found at higher altitudes and in the northern half of the island − which are generally cooler than those in which the species currently occurs. These uncertainties are further intensified by the availability of built environments in many parts of the island, which could help the species overcome macroclimatic constraints. Finally, climate change adds an extra layer of complexity to the estimation of suitable areas, as it will possibly contribute to a progressive reduction of climatic constraints imposed by low temperatures.

Species distribution models are a common and useful approach to predict the potential distribution of invasive species and disease vectors [14,15], including *Ae*. *aegypti* [3,16,17]. These models test for statistical relationships between species distribution data and spatial variables, aiming to identify the factors that determine observed species distributions and to predict the geographical distribution of suitable areas [18]. Some studies have used this approach to predict the distribution of suitable climates for *Ae*. *aegypti* at the global scale, which included Madeira Island [e.g.^19^,^20^]. However, a detailed assessment of suitable areas jointly considering climatic factors and fine scale patterns of land use and human settlement across the island is still lacking.

Here, we predict the distribution of suitable climates for *Ae*. *aegypti* on Madeira Island under current and future conditions, and jointly analyze these predictions with estimates of the suitability of land use and human settlement conditions. Our results contribute to understanding of the current and future potential distribution of this important diseases vector, and provide fundamental knowledge to support decision-making in the planning of monitoring and surveillance efforts.

## 2. Data and methods

### 2.1. Study area

**Fig 1:**
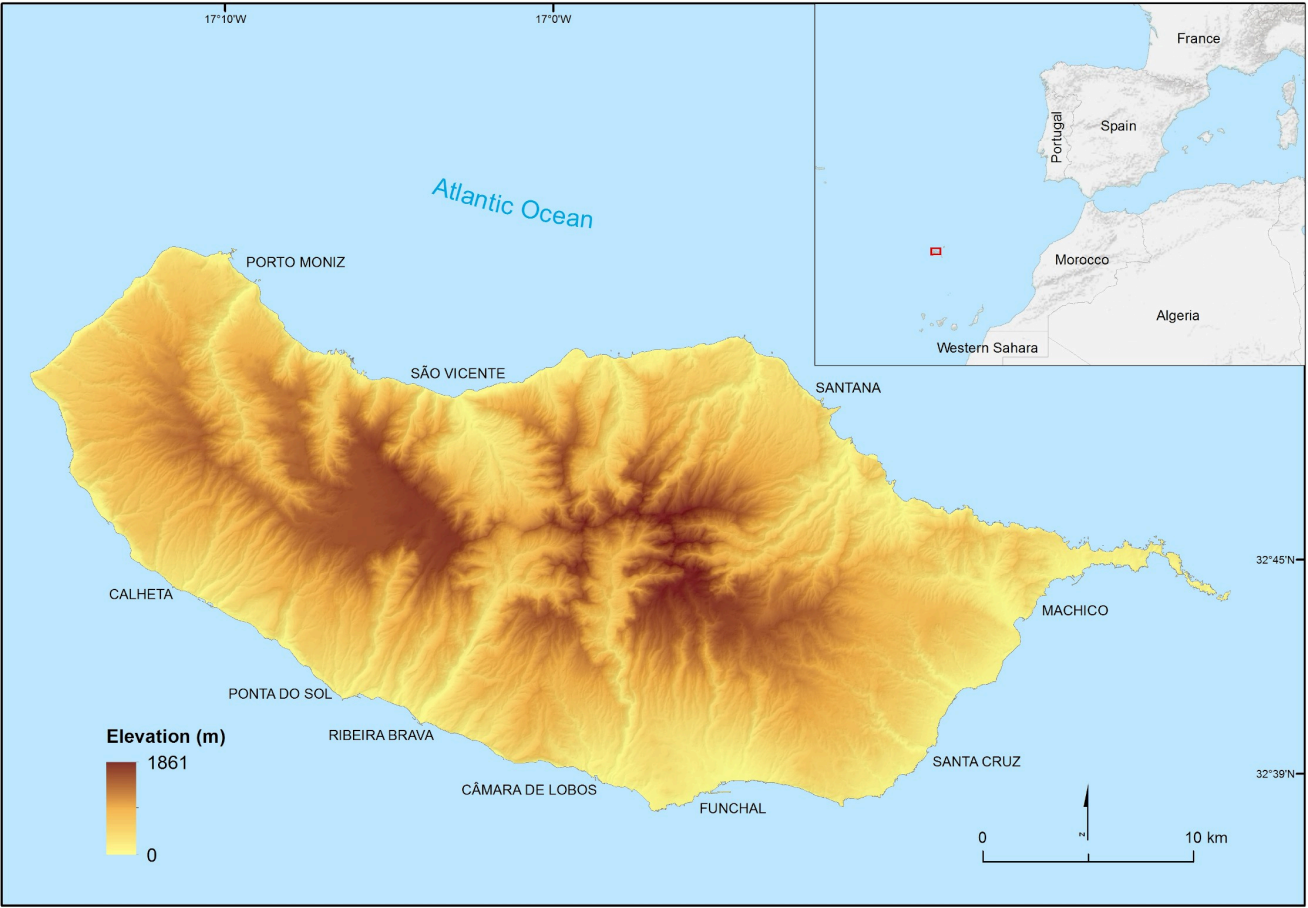
Geographical location and topography of Madeira Island (sources of shapefiles:https://www.dgterritorio.gov.pt/cartografia/cartografia-tematica/caop; and https://gadm.org/download_world.html).

The Island of Madeira is situated in the North Atlantic Ocean, approximately 600 km northwest of the West African coast and 850 km to mainland Portugal (Fig 1). The island has an area of approximately 740 km^2^ and its shape is elongated E-W (58 km long and 23 km wide). The maximum altitude is 1,861 m and about 90% of its area is over 500 m above sea level. The climate is generally mild, with average temperatures of around 18°C in winter and 25°C in summer. Temperatures above 30°C in summer are rare, and temperatures below 15°C in winter are generally restricted to higher altitudes areas. The main weather patterns show strong altitudinal and north-south asymmetry, with higher altitude areas and north-facing regions being considerably cooler and wetter [21].

### 2.2. Species distribution data

**Fig 2:**
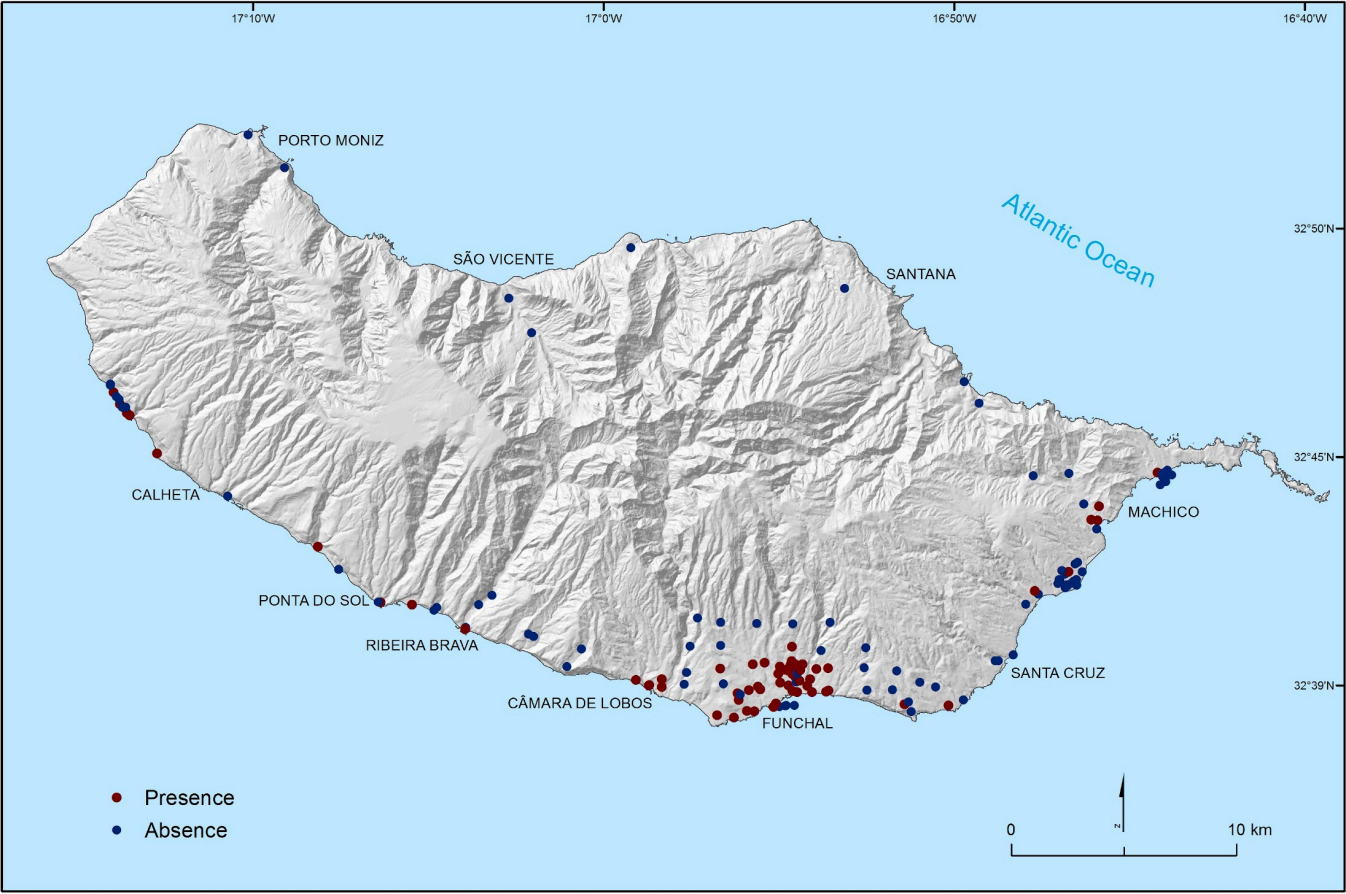
Spatial distribution of mosquito traps existing along the island and recorded presence or absence of *Aedes aegypti* mosquitoes (source of shapefile: https://www.dgterritorio.gov.pt/cartografia/cartografia-tematica/caop).

We obtained data on the distribution of the species on the island from the Madeira health authority–Madeira’s Regional Directorate for Health (DRS). These data come from a trap-based monitoring campaign conducted along the island since 2007 and consists of a total of 147 unique spatial records describing the distribution of the species up to 2017. In total, 61 records report the species presence and 86 its absence (Fig 2).

These data represent a small subset of the distribution of the species worldwide, which is unlikely to represent the full range of climatic conditions suitable to its establishment [22,23]. Thus, to obtain an improved representation of the species’ climatic niche [24,25], we combined the occurrence records in Madeira with occurrence records from around the world as provided by the Global Biodiversity Information Facility (GBIF) [26]. Records with a spatial precision lower than 1 km (the spatial resolution of climatic predictors; see below) were excluded. To avoid over-representation of environmental conditions in densely sampled areas [27], we kept only one observation record per 1 km grid cell. Thus, from the initial 41,568 occurrences obtained from both sources, only 10,614 were retained for model development (S1 Fig).

### 2.3. Climatic predictors

We collected global-scale climatic data at a resolution of 30 arc-second (c. 1km) from CHELSA (‘Climatologies at high resolution for the earth’s land surface areas’) database [28]. This is the highest spatial resolution available for climatic data covering the whole island.

Based on known eco-physiological requirements of the species, namely its sensitivity to extreme temperatures and water availability requirements [29], we selected seven variables to represent current climate conditions (1979–2013): i) mean temperature of the warmest quarter; ii) mean temperature of the coldest quarter; iii) maximum temperature of the warmest month; iv) minimum temperature of the coldest month; v) annual precipitation; vi) precipitation of the wettest quarter; and vii) precipitation of the driest quarter.

To assess the climatic suitability to the species under scenarios of future climates, we collected the same set of variables for the period 2041–2060. Three scenarios of future conditions were considered, representing radiative forcing values in the year 2100 of 2.6 W/m^2^, 4.5 W/m^2^ and 8.5 W/m^2^ (i.e., Representative Concentration Pathways 2.6, 4.5, and 8.5, respectively; ‘RCP26’, ‘RCP45’ and ‘RCP85’). Of these, RCP45 represents a so-called ‘intermediate’ scenario of greenhouse gas emissions that is expected to lead to an average increase of 1.4°C in global temperature by the mid of the century [30]. The RCP26 and RCP85, represent, respectively, an ‘optimistic’ and a ‘worst-case’ scenario, the first leading to an average increase of 1°C by the mid of the century and the second to an average increase of 2° C [30]. To account for variability among climate projections coming from distinct general circulation models (GCMs) [31], these variables were obtained by averaging the projections of five GCMs with the least amount of interdependency: CESM1_BCG, MPI_ESM_MR, MIROC5, CMCC-CM, CESM1_CAM [32].

### 2.4. Land use and human settlement predictors

We assembled a second set of variables representing patterns of land use and human settlement across the island. These factors are expected to shape the distribution of *Ae*. *aegypti* at fine scales, namely by determining the number of blood-feeding opportunities on humans [33], the availability of natural and artificial water containers used for oviposition (e.g. ornamental gardens, plant pots and fountains) [13] and the availability of infrastructures and habitats supplying shelter from extreme temperatures [34].

To represent these factors, we obtained land use data from the Regional Administration of Environment and Climate Changes (DRAAC) of the Madeira Government and data on population density and residential housing from Statistics Portugal (INE). These data were supplied in a vectorial structure with a minimum mapping width of 1 hectare.

The land use data has a hierarchical nomenclature, which at the highest level of disaggregation represents 100 classes. We merged these classes into 13 main urban, natural, and semi-natural environments present on the island, specifically: agricultural and agroforestry areas; artificial territories; banana plantations; bushes; low-rising continuous urban fabric; high-rising continuous urban fabric; sparse urban fabric; public and private artificial equipment; urban green areas; natural and semi-natural environments and water bodies (S2 Fig). Then for each mosquito trap on the island (Fig 2), we calculated the percentage of area occupied by each land use type within a 100m radius, a distance that reflects the usual flight dispersal of *Ae*. *aegypti* [35,36].

We also measured levels of human population density and residential housing in the surrounding of each trap. These measurements required the downscaling of the vectorial data, which are supplied at the level of census blocks. Census blocks correspond to city blocks in urban areas whereas in non-urban areas they can have wider spatial extents and comprise (or not) urban areas. Thus, the geographical boundaries of census blocks do not reflect the precise distribution of housing and people. To obtain an improved representation of these variables, we downscaled both by means of dasymetric mapping [37]. This technique is based on the identification of areas within census blocks that are populated (e.g. residential areas) and of areas that are not (e.g. green areas), and consists of using ancillary land use data to guide this spatial disaggregation [38]. We performed this operation by reclassifying land use data into urban and non-urban areas and then assigning the human population density and residential housing area in census blocks according to the relative weight of the disaggregated urban area. After this procedure, we measured the mean density of human population and of residential housing within a 100m radius of each trap.

### 2.5. Modelling procedures

#### 2.5.1. Predictions of climatic suitability

To assess the potential distribution of *Ae*. *aegypti* on the island, we started by developing predictions of climatic suitability. We developed the models at the global scale, using occurrence data of the species on the island and in other regions of the world as supplied by GBIF (see section 2.2, above).

Prior to the calibration of models, we used the variance inflation factor (VIF), as implemented in the *vif* function of R package usdm [39] to test for multicollinearity among the climatic variables. Using a VIF threshold of 10 [40], four non-redundant variables were retained: minimum temperature of coldest month, mean temperature of wettest quarter, precipitation of wettest quarter and precipitation of driest quarter.

Four algorithms available in the ‘sdm’ R package [41] were used to make the predictions: boosted regression trees (BRT), generalised additive models (GAM); generalised linear models (GLM) and random forests (RF). We used these four algorithms because they are among the most widely used and tested for species distribution modeling, performing well in various geographical contexts, scales of analysis, and with different species [42–44].

Each model was calibrated with climatic conditions represented by the species occurrence records and by a set of 11,000 pseudo-absences records randomly generated worldwide. The use of pseudo-absence data is a common practice to overcome the unavailability of true absence data [45] and our procedure followed recommended practices, by using a similar number of pseudo-absences and occurrence records [46]. Also, we extract pseudo-absences records across areas expected to have been available to the species [47], which for this cosmopolitan species consists of most of the world [48].

To evaluate the predictive performance of each model, we used a 5-fold cross-validation procedure with 10 repetitions [49]. In this procedure, the data is randomly divided into five equal-sized groups (‘folds’). Then four data folds are jointly used to calibrate each model ten times. This repetition is necessary to account for the stochasticity of machine learning algorithms, which can lead to slightly different predictions every time a new model calibration event is performed. Then, the left-out fold is used for comparison with the average of the predictions obtained from the 10 replicate models [14]. This procedure is repeated five times, each time using a distinct fold for comparison with the average predictions.

In addition to evaluating models at the global scale, we also evaluated their ability to predict the distribution of the species on the island. For this purpose, we calibrated climatic suitability models for each algorithm using all records of presence and pseudo-absence in other parts of the world. Each model was run ten times and its average prediction obtained. These predictions were then evaluated using the species occurrences in the island and an equal number of pseudo-absences randomly generated across the island (S3 Fig). We note that this assessment is likely less representative of the ’true’ accuracy of the models than the one described above, based on the range of species worldwide. This is because the traps, from which species occurrences were collected, are concentrated at lower altitudes, and do not cover well the climatic gradient existing on the island.

The predictive performance of models was assessed using two metrics: the area under the Receiver Operating Characteristic curve (AUC) and the true skill statistic (TSS) [50,51]. Values for these metrics above 0.8 and 0.5, respectively, are considered as representative of a ‘good’ predictive accuracy [52,53]. We also evaluated the relative influence of each predictor in the models through AUC-based permutations tests, as provided by the ‘getVarImp’ function of the ‘sdm’ R package.

Following these procedures, we applied the models for predicting climatic suitability on the island under current and projected conditions for 2041–2060.

#### 2.5.2. Predictions of suitability based on land use and human settlement patterns

We built a second set of models for predicting the suitability of land use and human settlement conditions across the island. This set of models used the species presence or absence in the network of traps existing along the island as response variable (Fig 2) and the variables representing land use and human settlement conditions as predictors (see section 2.4 above). In contrast to the climate suitability estimates, we expect the distribution data captured by the network of traps existing along the island to provide a reasonable representation of the suitability of land-use and human settlement factors. This is because land use and human settlement conditions vary widely over short distances (S2 Fig) and the diversity of conditions in these factors should be well sampled by these data. In addition, the traps are mainly located in areas where the species has already reached (S4 Fig), so the distribution pattern represented is not strongly affected by dispersal limitations [54].

As performed for the climatic suitability models, we evaluated multicollinearity among predictor variables prior to model calibration. No variable was identified as redundant. The predictive accuracy of models was also evaluated by means of a 5-fold cross-validation procedure with 10 repetitions. However, in this case we could not use the GAM algorithm, as the number of distribution records was insufficient for model fitting.

To perform predictions from these models, we built a grid of regular hexagons with c. 100m radius each, covering the whole island (S5 Fig). Each hexagon represented the values of percentage of land classes and average population and housing density–following the same procedures used for the predictor variables.

#### 2.5.3. Visualizing predictions of climatic suitability and of suitability of land use and human settlement conditions

To attain final estimates of the potential distribution of the species on Madeira Island, we created an ensemble of model predictions using the ‘frequency histogram’ approach [55]. This approach consists of overlaying and summing binary maps of suitable and unsuitable areas predicted by the different models in the ensemble. A high frequency of models predicting an area as suitable or unsuitable indicates greater inter-model consensus and therefore greater confidence in each of the results. Intermediate frequency values reflect disagreement between models and correspond to areas of higher uncertainty. For this purpose, we classified the individual predictions into suitable or unsuitable using the ‘10th percentile’ approach [56,57]. The ensembles included predictions from all models, as they achieved a ‘good’ accuracy at least one of metrics used (i.e., TSS > 0.5 or AUC > 0.8; see results). In the case of climatic suitability models, this accuracy was based the *k*-fold validation performed at the global scale, as this is expected to be more robust than the one performed for the island (see section 2.5.1. above). We visualized the overlay of predictions for climate only, land use and human settlement only and for the two sets of factors in combination.

## 3. Results

### 3.1. Performance of models

The evaluation of climatic suitability models performed at the global scale retrieved high AUC and TSS values, ranging between 0.91 and 0.97 and from 0.75 to 0.85, respectively. In the evaluation performed for the island, AUC varied between 0.92 and 0.62 and TSS from 0.9 to 0.44, with the lower values for each metric being recorded for GAM (S1 Table). Predictions of suitability based on land use and human settlement predictors also recorded marginally good accuracies, with AUC and TSS values ranging from 0.79 to 0.82 and 0.58 to 0.62, respectively (Table 1).

**Table 1:** Predictive performance of algorithms used in the prediction of climatic suitability and suitability of land use and human settlement patterns. The algorithms used were boosted regression trees (BRT), generalized additive models (GAM), generalized linear models (GLM) and random forests (RF). Generalized additive models were not used to assess suitability of land use and human settlement patterns as the number of distribution records was insufficient for model fitting.

	Climatic suitability				Suitability of land use and human-related factors		
	BRT	GAM	GLM	RF	BRT	GLM	RF
AUC	0.94	0.94	0.91	0.97	0.81	0.79	0.81
TSS	0.81	0.8	0.75	0.85	0.61	0.58	0.62

### 3.2. Relative influence of predictor variables and response curves

**Fig 3:**
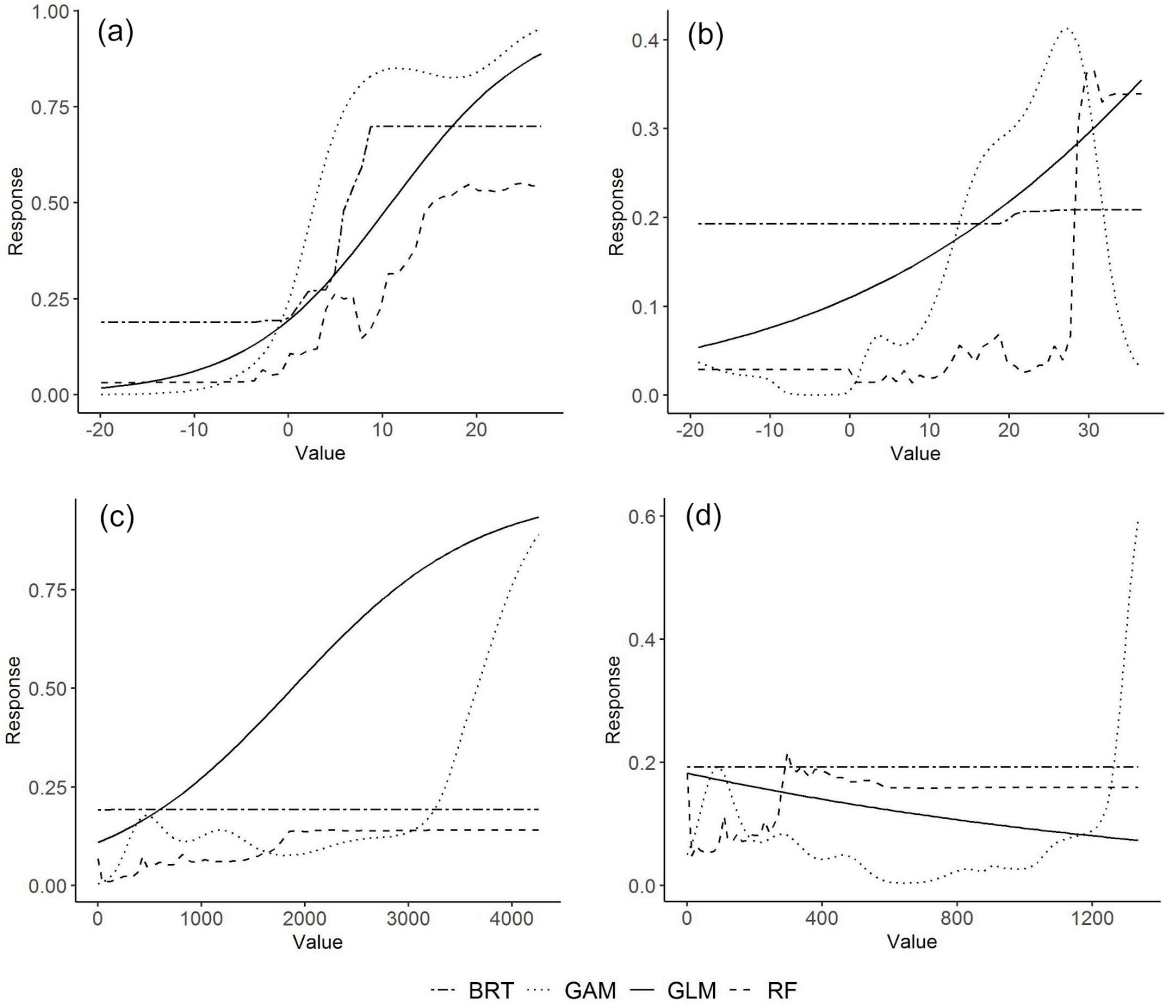
Response curves of predicted suitability for *Aedes aegypti* along the gradient of climatic predictor variables: a) minimum temperature of coldest month; b) mean temperature of wettest quarter; c) precipitation of wettest quarter; d) precipitation of driest quarter.

Minimum temperature of the coldest month was identified as the most influential variable in all climatic suitability models (BRT- 78.7; GAM- 57.8; GLM- 90.9 and RF- 54.8), mean temperature of wettest quarter (BRT- 0.2; GAM- 21.5; GLM-5.0; RF- 15.9) and precipitation of wettest quarter (BRT- 15.2; GAM- 17.9; GLM- 3.9; RF- 18.5) had intermediate levels of influence in some models and precipitation of driest quarter (BRT- 5.9; GAM- 2.8; GLM- 0.2; RF- 10.8) had a consistently low influence across models (Table 2). Response curves for the three most influential predictor variables indicate that, *Ae*. *aegypti* occurs mainly in areas where the minimum temperature in the coldest month and in the wettest quarter is above 0°C and in areas with abundant precipitation during the wettest quarter (Fig 3).

**Table 2:** Relative influence of predictor variables in models of climatic suitability for boosted regression trees (BRT), generalized additive models (GAM), generalized linear models (GLM) and random forests (RF).

	BRT	GAM	GLM	RF
Mean temperature of wettest quarter	0.2	21.5	5.0	15.9
Minimum temperature of coldest month	78.7	57.8	90.9	54.8
Precipitation of driest quarter	5.9	2.8	0.2	10.8
Precipitation of wettest quarter	15.2	17.9	3.9	18.5

**Fig 4:**
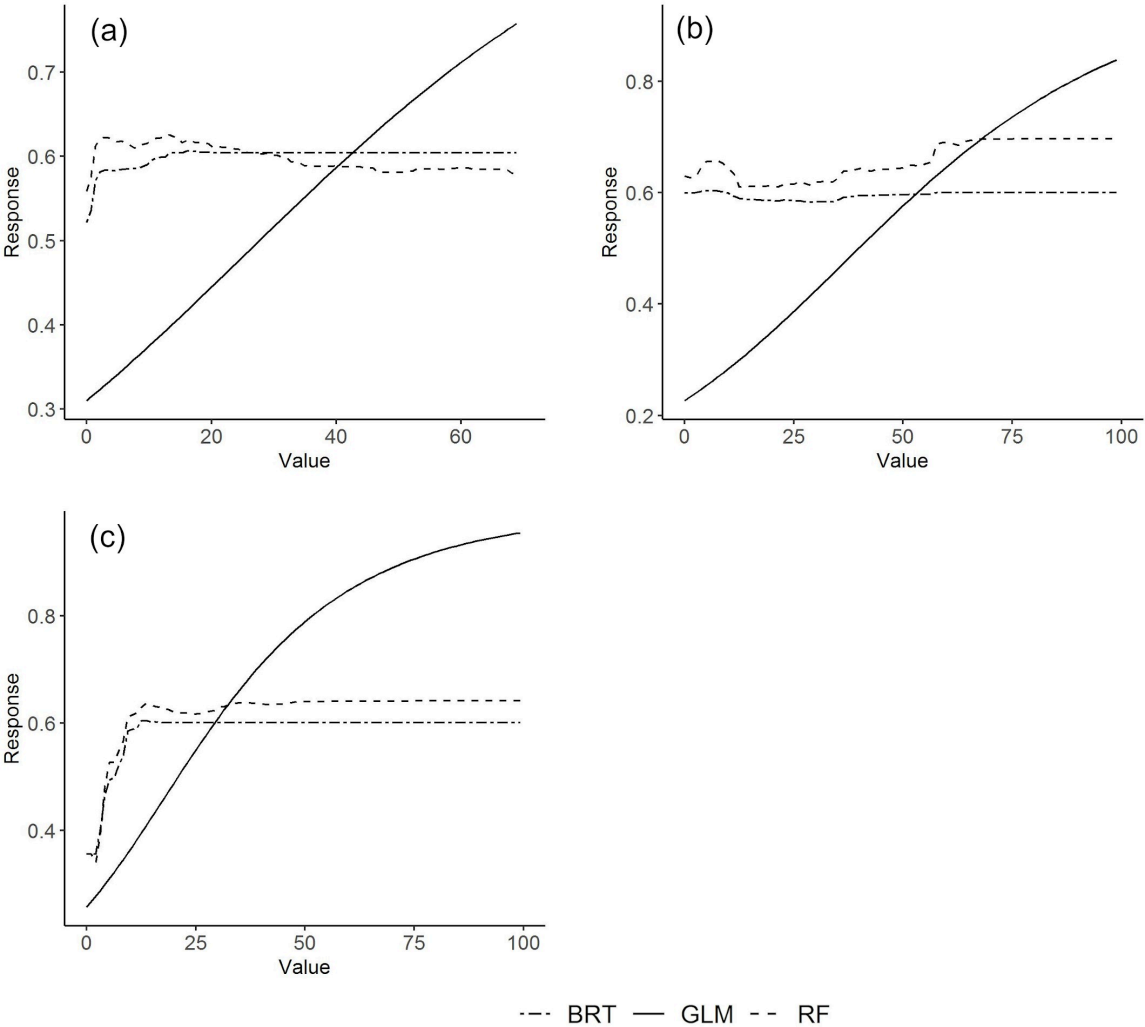
Response curves of predicted suitability for *Aedes aegypti* along the gradient of most influential predictor variables representing land use and human settlement conditions: a) percentage of area occupied by public equipment; b) residential house density; c) human population density. Generalized additive models were not used as the number of distribution records was insufficient for model fitting.

With respect to land use and human settlement patterns, three variables stand out in terms of their influence in the models: population density (BRT- 59.1; GLM-22.3; RF- 34.2), the land use class ‘public equipment’ (e.g. schools) (BRT- 22.1; GLM- 15.5; RF- 24.7), and residential house density (BRT- 4.5; GLM- 23.7; RF-12.3) (Table 3). Remaining variables had a modest to reduced influence, regardless of the modeling algorithm used. All three variables identified as most influential are positively linked to levels of suitability (Fig 4). For BRT and RF the increase in suitability levels only occurs up to one third to half of the gradient of the variables, after which suitability values remain constant.

**Table 3:** Relative influence of predictor variables in models of suitability of land use and human settlement conditions for boosted regression trees (BRT), generalized linear models (GLM) and random forests (RF). Generalized additive models were not used as the number of distribution records was insufficient for model fitting.

Environmental variables	BRT	GLM	RF
Agricultural areas and agro-forestry	3.4	11	3.9
Artificialized territories	1.7	4.6	2.2
Banana plantation	1.4	1.7	1.8
Bushes	1.5	0.6	3.2
Discontinuous urban fabric	0.2	0.9	0.9
Forests and natural environments	0.9	5.4	2
High-rising continuous urban fabric	0.9	1.7	2.5
Low-rising continuous urban fabric	4.1	8.1	7.7
Parks and gardens	0.2	0.8	2.4
Population density	59.1	22.3	34.2
Public equipment	22.1	15.5	24.7
Residential house density	4.5	23.7	12.3
Sparse discontinuous urban fabric	0	0.5	1.8
Water bodies	0	3.2	0.4

### 3.3. Predicted potential distribution

**Fig 5:**
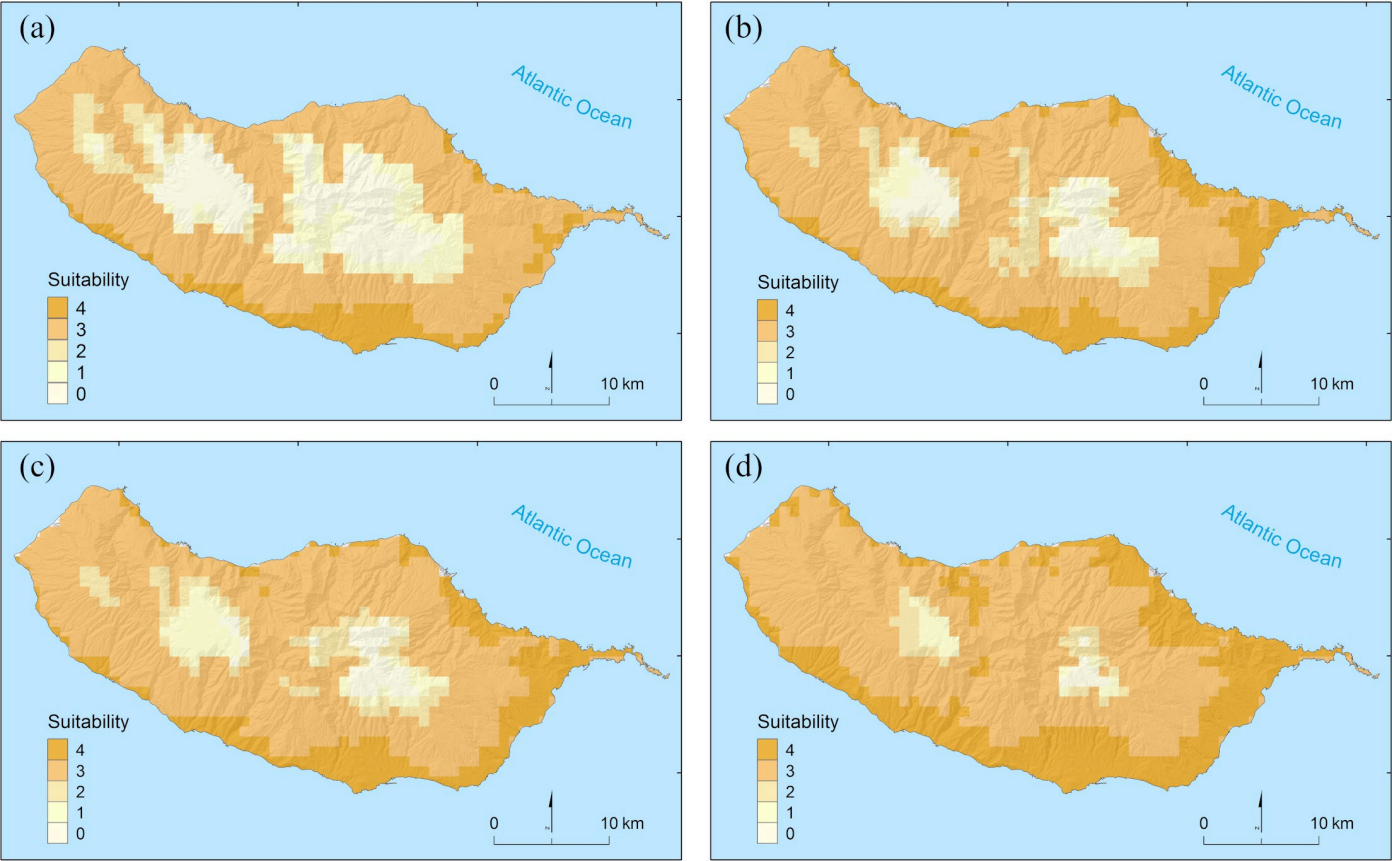
Inter-model consensus on climatic suitability for *Aedes aegypti* on Madeira Island. The highest (lowest) value indicates areas predicted as suitable (unsuitable) by all four algorithms (boosted regression trees, generalized linear models, generalized additive models and random forests) Intermediate values reflect disagreement between models and correspond to areas of higher uncertainty. Predictions are provided for current climatic conditions (a) and for climatic conditions projected for the mid of the 21^st^ century (2041–2060) under RCP2.6 (b), RCP4.5 (c) and RCP8.5 (d) (source of shapefile: https://www.dgterritorio.gov.pt/cartografia/cartografia-tematica/caop).

By overlaying the binary predictions of the four algorithms (see individual predictions in S6 Fig), we identify that inter-model consensus on current climatic suitability occurs for only 9.5% of the island (Fig 5A). These areas are found along the coast, mainly on the south side of the island. Three out of four algorithms predict wider extents of suitable climates, including at mid-altitudes and on the north side of the island (Fig 5A).

When considering the climate projected for by the mid of the century, all models suggest an increase in the extent of suitable areas (Fig 5B–5D). This increase is narrower under RCP2.6 (Fig 5B) and higher under RCP8.5 (Fig 5D). In comparison to current conditions, the areas predicted as climatically suitable by all models under RCP4.5 nearly doubles (16.9% of the island). Under RCP2.6 the increase in extent is reasonably similar (15.8%), while under RCP8.5 is substantially higher (31.6%) (see individual predictions in S7–S9 Figs).

**Fig 6:**
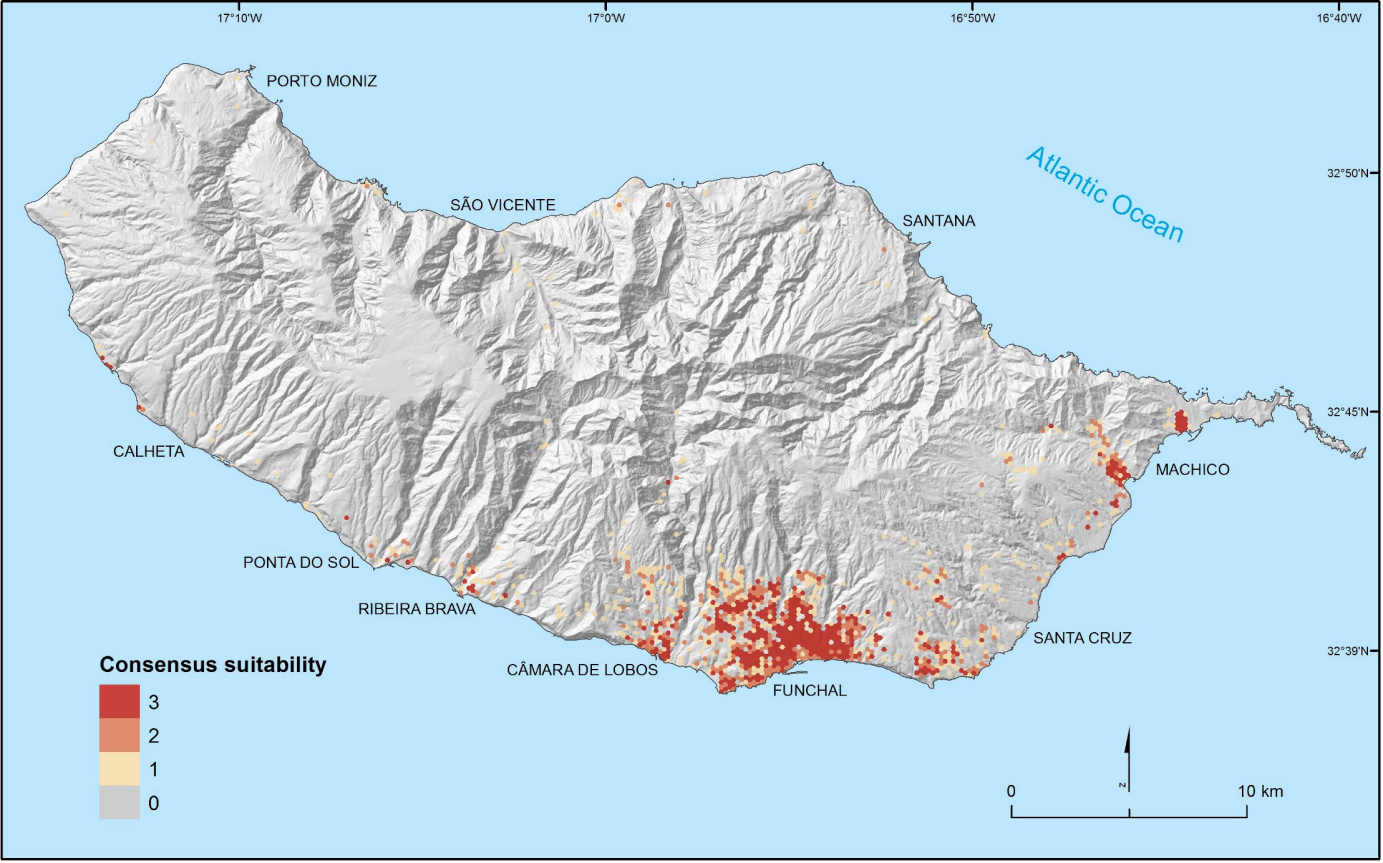
Suitability of land use and human settlement conditions for *Aedes aegypti* on Madeira Island. The highest (lowest) value indicates areas predicted as suitable (unsuitable) by all three algorithms: boosted regression trees, generalized linear models and random forests (generalized additive models were not used as the number of distribution records was insufficient for model fitting). Intermediate values reflect disagreement between models and correspond to areas of higher uncertainty on the suitability of conditions (source of shapefile: https://www.dgterritorio.gov.pt/cartografia/cartografia-tematica/caop).

Concerning land use and human settlement patterns, inter-model consensus on suitable areas (i.e., predicted as suitable by all three algorithms used) occurs mainly for urban areas located on the south coast of the island (Fig 6) (see individual predictions in S10 Fig). Areas predicted as suitable by at least two algorithms expand the previous areas to locations in their surroundings including some inhabited areas located further into the interior of the island and at higher elevations. A few, small-sized, isolated patches on the northern coast of the island are also identified as suitable.

**Fig 7:**
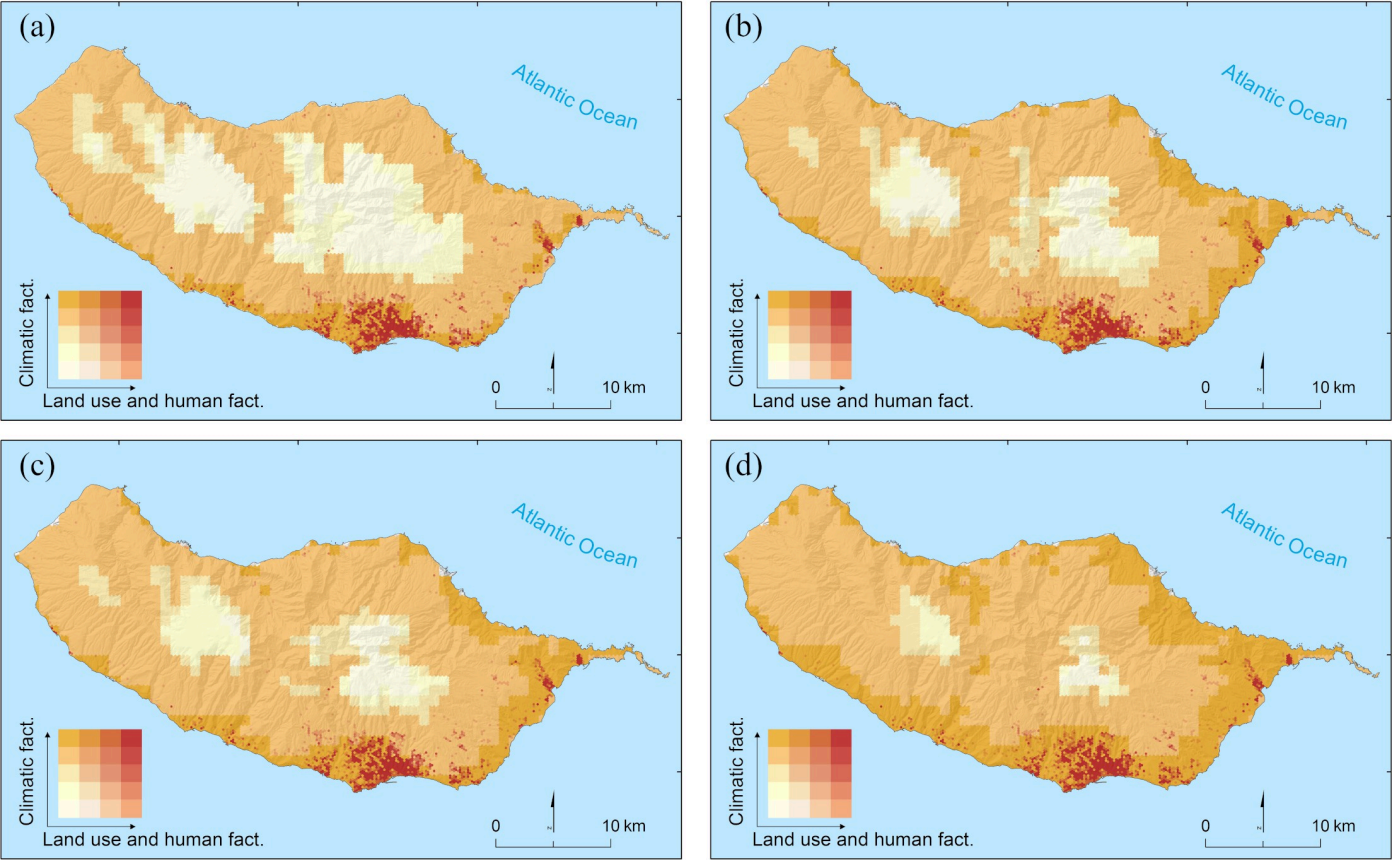
Suitability of climatic and of human-related or land use conditions for *Aedes aegypti* on Madeira Island. Inter-model consensus on suitable conditions for each set of factors is represented along a bivariate gradient, where darker (lighter) colors on each axis represent higher (lower) consensus on suitable conditions. Estimates of climatic suitability are provided for current conditions (a) and the mid of the 21st century (2041–2060) under RCP2.6 (b), RCP4.5 (c) and RCP8.5 (d) (source of shapefile: https://www.dgterritorio.gov.pt/cartografia/cartografia-tematica/caop).

Finally, the combined examination of consensus for current climate and land use and human settlement conditions identifies only 1.6% of the island as suitable with high certainty, i.e., predicted as suitable by all models for the two types of factors (Fig 7A). These areas are concentrated in the southern and north-eastern coastal strip of the island (Fig 7A); the former being already largely colonized by the species (Fig 2). On the other hand, no model, regardless of the type of predictor variables, predicts suitable conditions for 16.2% of the island, corresponding mainly to the areas at higher altitudes. The joint analysis of predictions for the two types of factors shows that 86% of the area predicted as suitable with high certainty in terms of land use and human settlement patterns occurs in areas where there is also high certainty on climatic suitability. However, this overlap accounts for only 16.5% of the area of the latter, meaning that most climatically suitable areas do not offer suitable human-related or land use conditions. Assuming unchanging conditions of land use and human settlement patterns, the extent of areas simultaneously suitable in terms of climatic and human and land-use factors will remain relatively similar by the mid of the century, as climatic suitability will mainly emerge in areas without suitable human-related or land use conditions. Specifically, these areas will correspond to 1.5%, 1.6% and 1.8% of the island under RCP2.6, RCP4.5 and RCP8.5, respectively (Fig 7B–7D).

## 4. Discussion

In this work, we used a species distribution modelling approach to assess the current and future potential distribution of *Ae*. *aegypti* on Madeira Island. We have considered both climatic and land use and human-related factors, for which we have developed independent but complementary predictions of suitability. Both types of factors delivered models that predicted the distribution of the species with overall good accuracy, supporting the robustness of the suitability estimates obtained.

Under current conditions, areas predicted as climatically suitable by all algorithms (i.e., those of higher inter-model consensus) are concentrated in the north-eastern and southern coastal strips of the island. The number of models predicting areas as climatically suitable decreases progressively towards the interior of the island as altitude increases, apparently reflecting the thermal geography of the island − where warmer conditions are found closer to the ocean, particularly in the southern half of the island [21]. Nevertheless, it is noteworthy the occurrence of wider areas predicted as suitable by three (out of four) algorithms, mainly at mid-altitudes. The temperatures found in these areas are likely close to the cold tolerance limit of the species and for which a precise delimitation is difficult to achieve through statistical approaches [58,59]. However, given the support provided by most of our models, it seems plausible that populations of the species may occur in some of these areas, even if only temporarily or conditional on the availability of favorable land-use and human settlement conditions (see below).

Models based on land use and human settlement conditions predicted, with overall good accuracy, the distribution of species in the network of traps existing along the island. This result likely benefits from most traps being located at low elevations, where climates are generally suitable, thus accentuating the role of non-climatic factors as drivers of the distribution sampled. These models also identified a positive association between the species occurrence and areas with higher density of residential housing, public spaces, and human population, reflecting the known association of the species with built environments and densely populated areas [13]. The favorability of these areas for the species is also apparent in the island. For example, the species’ use of peri-domiciliary water storage containers (related to the presence of ornamental gardens, vases, etc.), led to a public campaign aimed at avoiding the availability of these containers [8]. Regarding public spaces (e.g. schools), the regular presence of human activity, despite the low number of residents, as well as the existence of potential breeding sites (e.g. small gardens and disposable objects resulting from dumping) may explain a higher probability of occurrence of mosquitoes in these places.

Focusing on areas predicted to have simultaneously suitable climates and suitable land use and human-related factors, our results suggest that *Ae*. *aegypti* already colonized most of its current potential distribution on the island. This is not surprising given the relatively narrow and spatially circumscribed distribution of these areas, and that the species has also been able to disperse widely across the island since its first detection [6,7]. Moreover, our results also show that while areas with suitable climates are expected to become wider as time progresses (mainly in the north-eastern and south-eastern parts of the island), most of these newly suitable areas will emerge where land use and human-related conditions are unfavorable, likely limiting the range expansion of the species. However, it is important to note that our assessment is based on the maintenance of current patterns of land use or human settlement. Thus, divergent future trajectories with respect to these factors will likely lead to different outcomes. For example, an increase in the species range is likely to occur if urban areas expand into these new climatically suitable areas or if new public infrastructures are built there.

It would be valuable if some limitations of our assessments could be overcome in future work. A relevant improvement would be the implementation of climatic suitability models at a higher spatial resolution. Currently this is not feasible, as these models need to consider the worldwide distribution of the species to ensure a comprehensive representation of its climate niche, and at this extent the most detailed climate data available are provided at the resolution we use. In this respect, one possibility for improvement could be to downscale the climate suitability projections [60], or the climate variables themselves [61]. However, these procedures may also imply an increase in errors and uncertainty in the forecasts [62], going against the aim of our work. Another limitation concerns the assumption of niche conservatism that underlies our models, as they do not consider adaptive changes in the species’ ecological tolerances [63]. While previous work has identified the stability of the climatic niche of *Ae*. *aegypti* across invaded regions worldwide [64] − supporting the robustness of the predictions we obtained − future work could explicitly consider the potential for adaptative changes by means of mechanistic models accounting for evolutionary responses [63].

We provide detailed estimates of the potential distribution of *Ae*. *aegypti* on Madeira Island. Our results suggest that both climatic factors and patterns of land use and human settlement interact to shape the current distribution of the species on the island and that most currently suitable conditions are already colonised by the species. However, we also find that as time progresses, climatically suitable areas will expand, particularly in the north-eastern and south-eastern regions of the island. The implications of these increases will need to be considered in conjunction with future land use and human settlement patterns and point to the need to increasingly consider the effects of climate change in ongoing efforts to prevent and control the spread of the species on the island.

## Supporting information

S1 Table Predictive performance of algorithms used in the prediction of climatic suitability. Model evaluation is based on the known distribution of the species on the island and an equal number of random pseudo-absence records. The algorithms used were boosted regression trees (BRT), generalized additive models (GAM), generalized linear models (GLM) and random forest (RF). Predictive performance was measured by means of the area under the Receiver Operating Characteristic Curve (AUC) and of the true skill statistic (TSS).
(DOCX)

S1 Fig Global distribution of *Aedes aegypti* occurrences used to develop the climatic models (source of shapefile: https://gadm.org/download_world.html). (TIF)

S2 Fig Land use map for Madeira Island (source of land use data: Madeira’s Regional Administration of Environment and Climate Changes; source of shapefile: https://www.dgterritorio.gov.pt/cartografia/cartografia-tematica/caop). (TIF)

S3 Fig Spatial distribution of species occurrences and pseudo-absences randomly generated across Madeira Island (one record per ~1 km grid cell) (source of shapefile: https://www.dgterritorio.gov.pt/cartografia/cartografia-tematica/caop). (TIF)

S4 Fig Year of the first record of *Aedes aegypti* on municipalities of Madeira Island up to 2015 (source of data: Madeira’s Regional Directorate for Health; source of shapefile: https://www.dgterritorio.gov.pt/cartografia/cartografia-tematica/caop). (TIF)

S5 Fig Hexagonal grid used to perform the local predictions (source of shapefile: https://www.dgterritorio.gov.pt/cartografia/cartografia-tematica/caop). (TIF)

S6 Fig Predictions obtained for distinct modelling algorithms under current climatic conditions BRT(a), GAM (b), GLM (c) an RF (d) (source of shapefile: https://www.dgterritorio.gov.pt/cartografia/cartografia-tematica/caop).
(TIF)

S7 Fig Predictions obtained for distinct modelling algorithms based on climatic conditions projected for the mid of the 21^st^ century (2041–2060) under RCP2.6 BRT(a), GAM (b), GLM (c) an RF (d) (source of shapefile: https://www.dgterritorio.gov.pt/cartografia/cartografia-tematica/caop).
(TIF)

S8 Fig Predictions obtained for the distinct modelling algorithms based on climatic conditions projected for the mid of the 21st century (2041–2060) under RCP4.5 BRT(a), GAM (b), GLM (c) an RF (d) (source of shapefile: https://www.dgterritorio.gov.pt/cartografia/cartografia-tematica/caop).
(TIF)

S9 Fig Predictions obtained for the distinct modelling algorithms based on climatic conditions projected for the mid of the 21st century (2041–2060) under RCP8.5 BRT(a), GAM (b), GLM (c) an RF (d) (source of shapefile: https://www.dgterritorio.gov.pt/cartografia/cartografia-tematica/caop).
(TIF)

S10 Fig Predictions obtained for the distinct modelling algorithms based on land use and human settlement conditions (source of shapefile: https://www.dgterritorio.gov.pt/cartografia/cartografia-tematica/caop). (TIF)
