# Supplementary material for: The current and future distribution of the yellow fever mosquito (Aedes aegypti) on Madeira Island
Source: PLoS Negl Trop Dis. 2022 Sep 12;16(9):e0010715. doi: 10.1371/journal.pntd.0010715 (PMC9499243; doi:10.1371/journal.pntd.0010715)
Supplement: S1 Table — Model evaluation is based on the known distribution of the species on the island and an equal number of random pseudo-absence records. The algorithms used were boosted regression trees (BRT), generalized additive models (GAM), generalized linear models (GLM) and random forest (RF). Predictive performance was measured by means of the area under the Receiver Operating Characteristic Curve (AUC) and of the true skill statistic (TSS). (DOCX) [file pntd.0010715.s001.docx]

|  | BRT | GAM | GLM | RF |
| --- | --- | --- | --- | --- |
| AUC | 0.92 | 0.62 | 0.91 | 0.91 |
| TSS | 0.9 | 0.44 | 0.87 | 0.77 |
