# Supplementary figures and images for: The current and future distribution of the yellow fever mosquito (Aedes aegypti) on Madeira Island
Source: PLoS Negl Trop Dis. 2022 Sep 12;16(9):e0010715. doi: 10.1371/journal.pntd.0010715 (PMC9499243; doi:10.1371/journal.pntd.0010715)

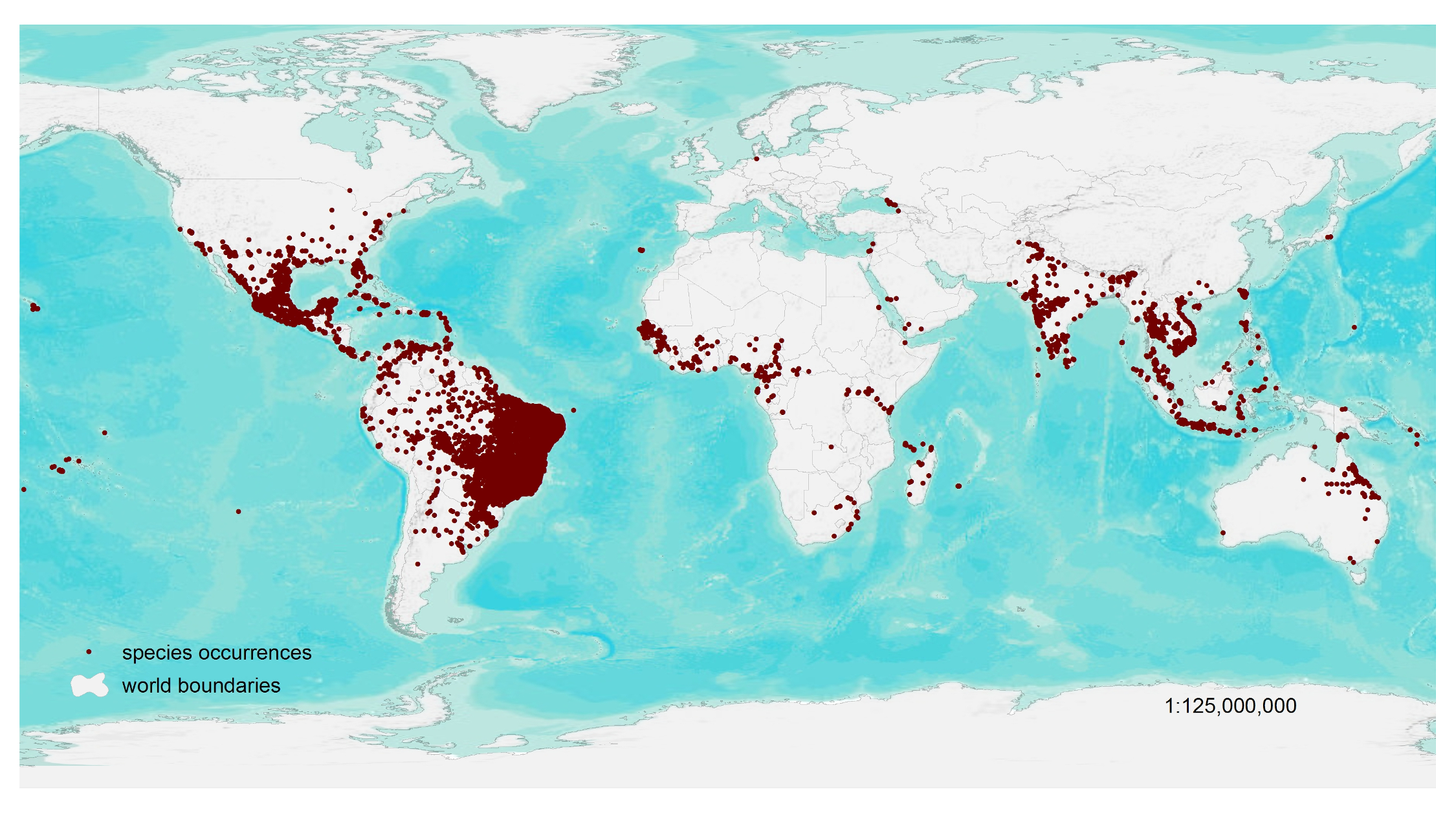

Supplement: S1 Fig — (TIF) [file pntd.0010715.s002.tif]

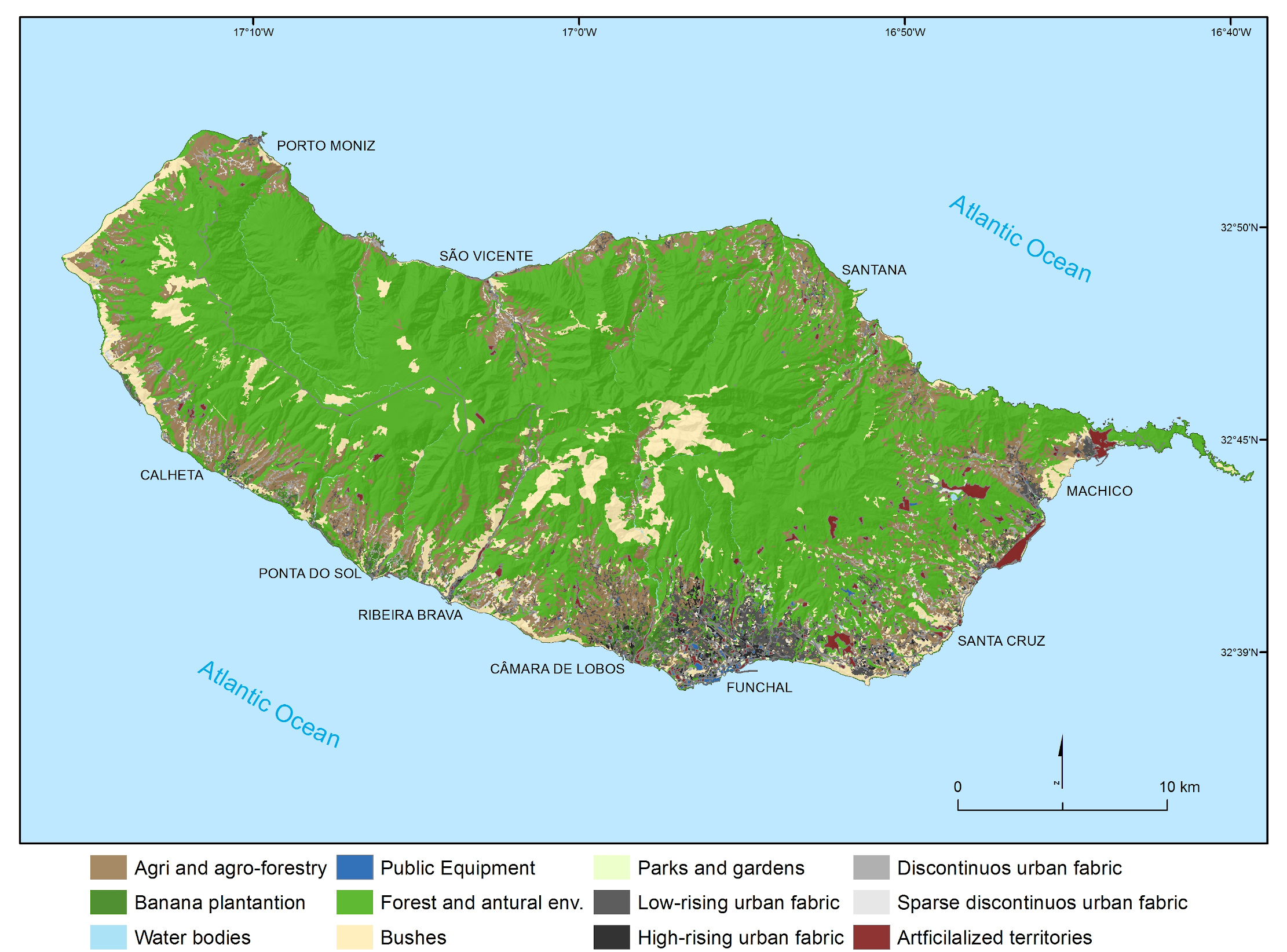

Supplement: S2 Fig — (TIF) [file pntd.0010715.s003.tif]

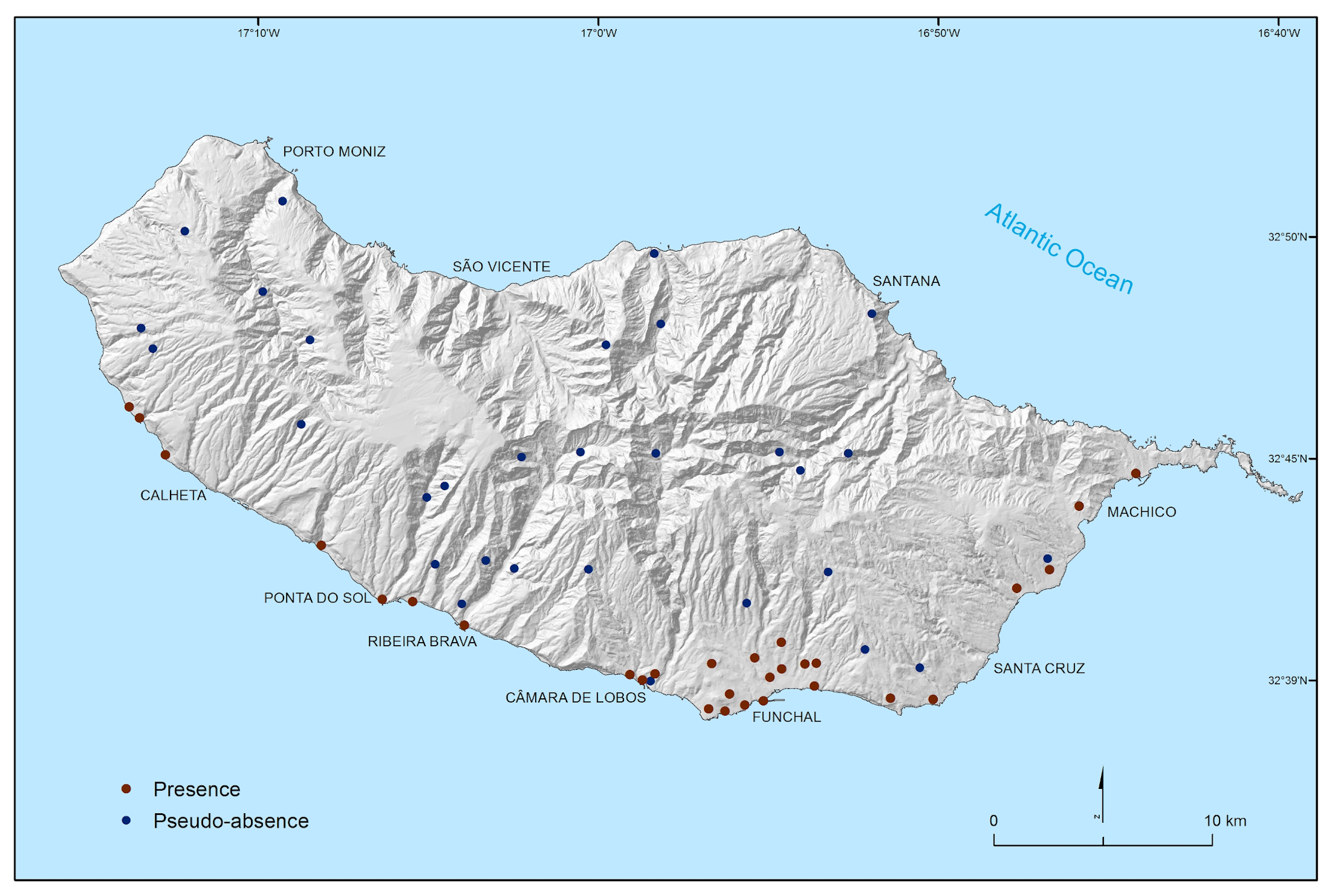

Supplement: S3 Fig — (TIF) [file pntd.0010715.s004.tif]

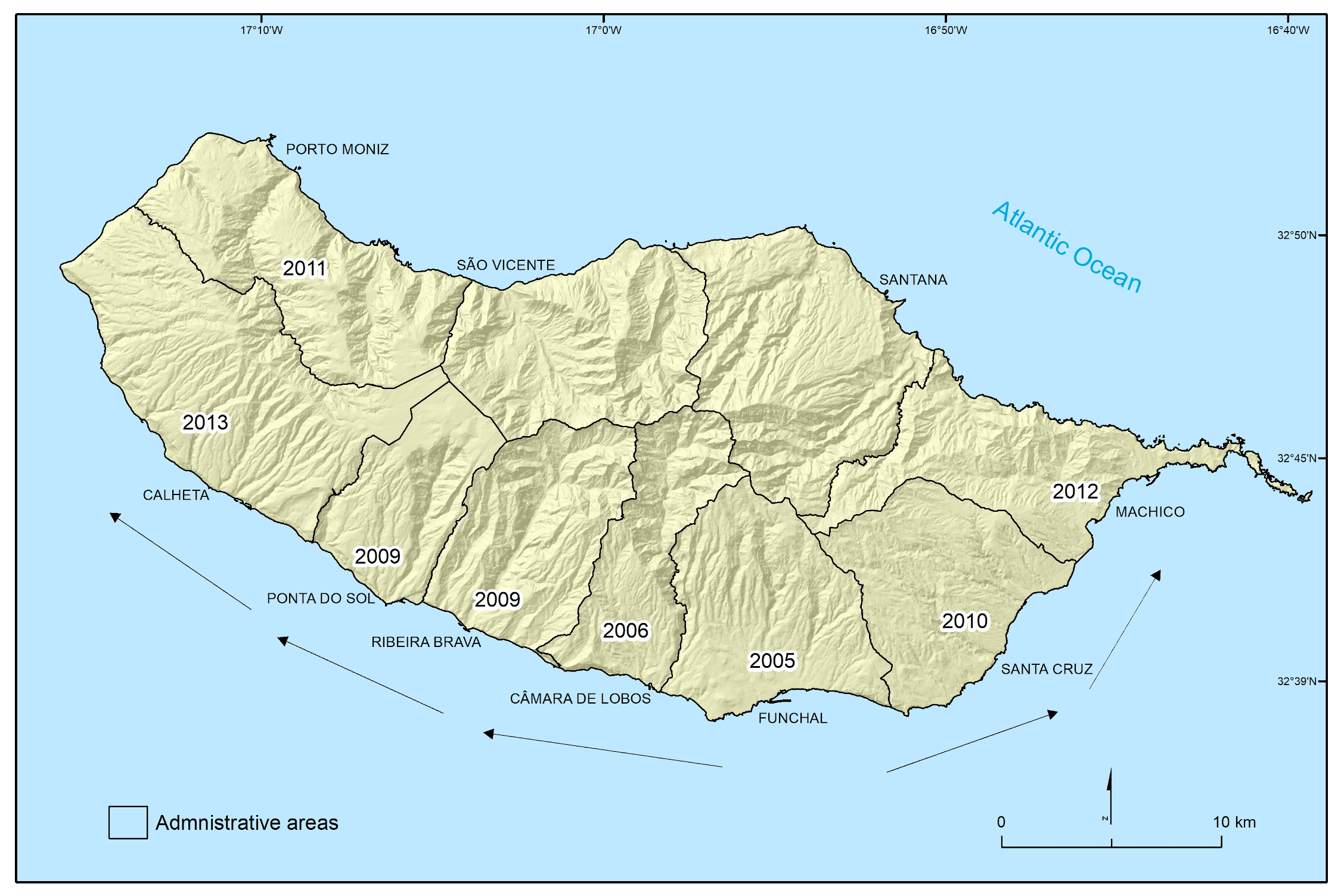

Supplement: S4 Fig — (TIF) [file pntd.0010715.s005.tif]

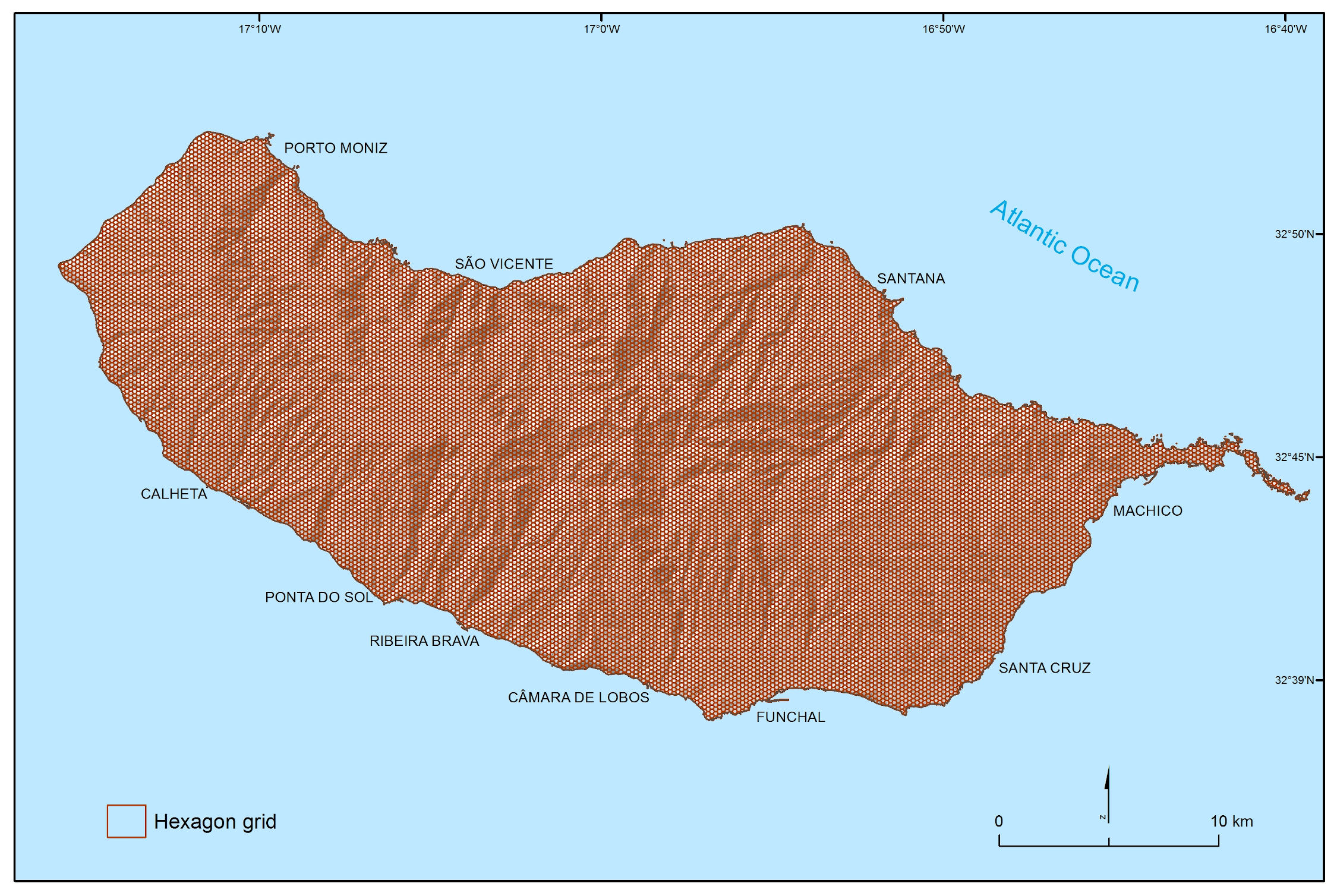

Supplement: S5 Fig — (TIF) [file pntd.0010715.s006.tif]

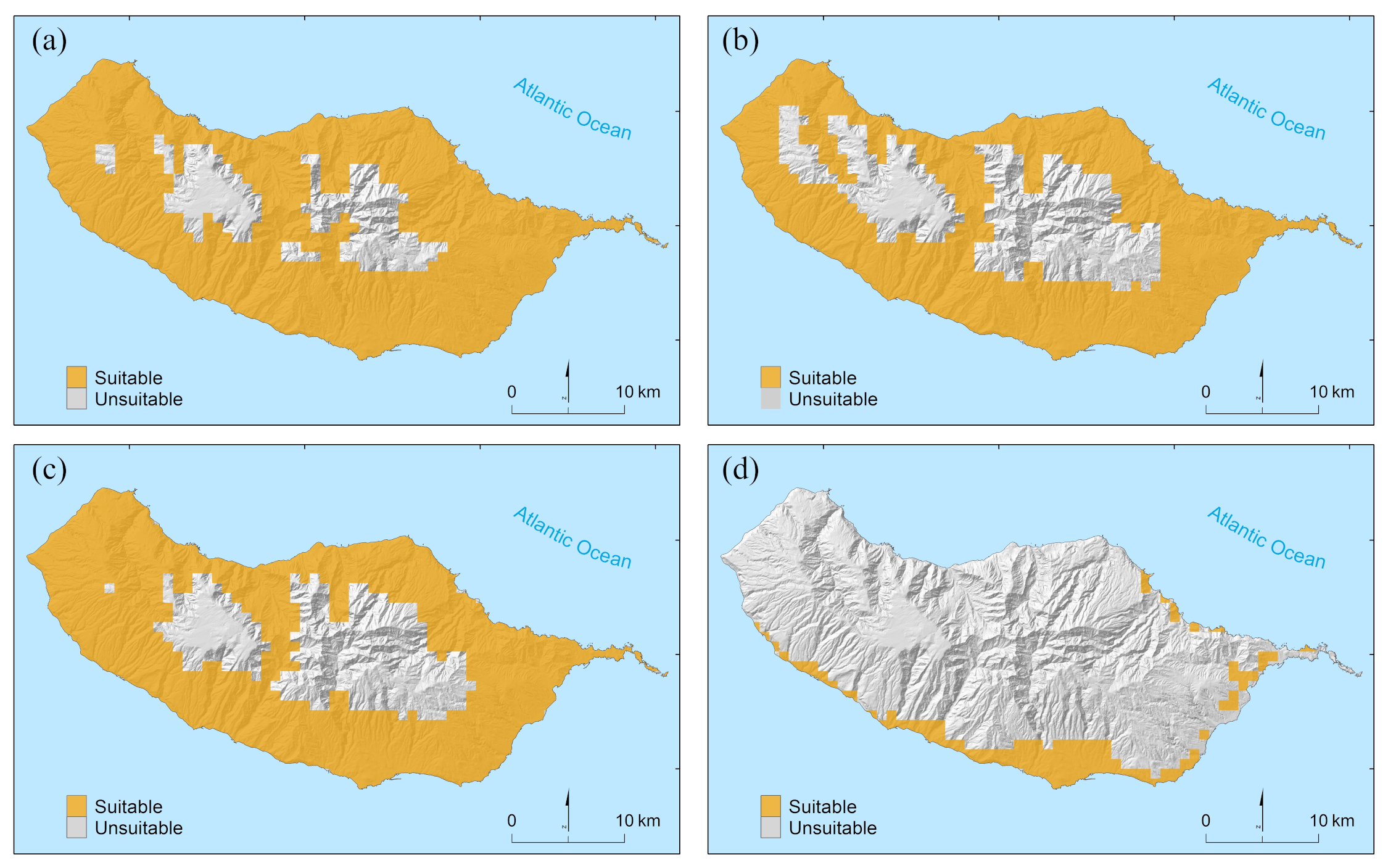

Supplement: S6 Fig — Predictions obtained for distinct modelling algorithms under current climatic conditions BRT(a), GAM (b), GLM (c) an RF (d) (source of shapefile: https://www.dgterritorio.gov.pt/cartografia/cartografia-tematica/caop). (TIF) [file pntd.0010715.s007.tif]

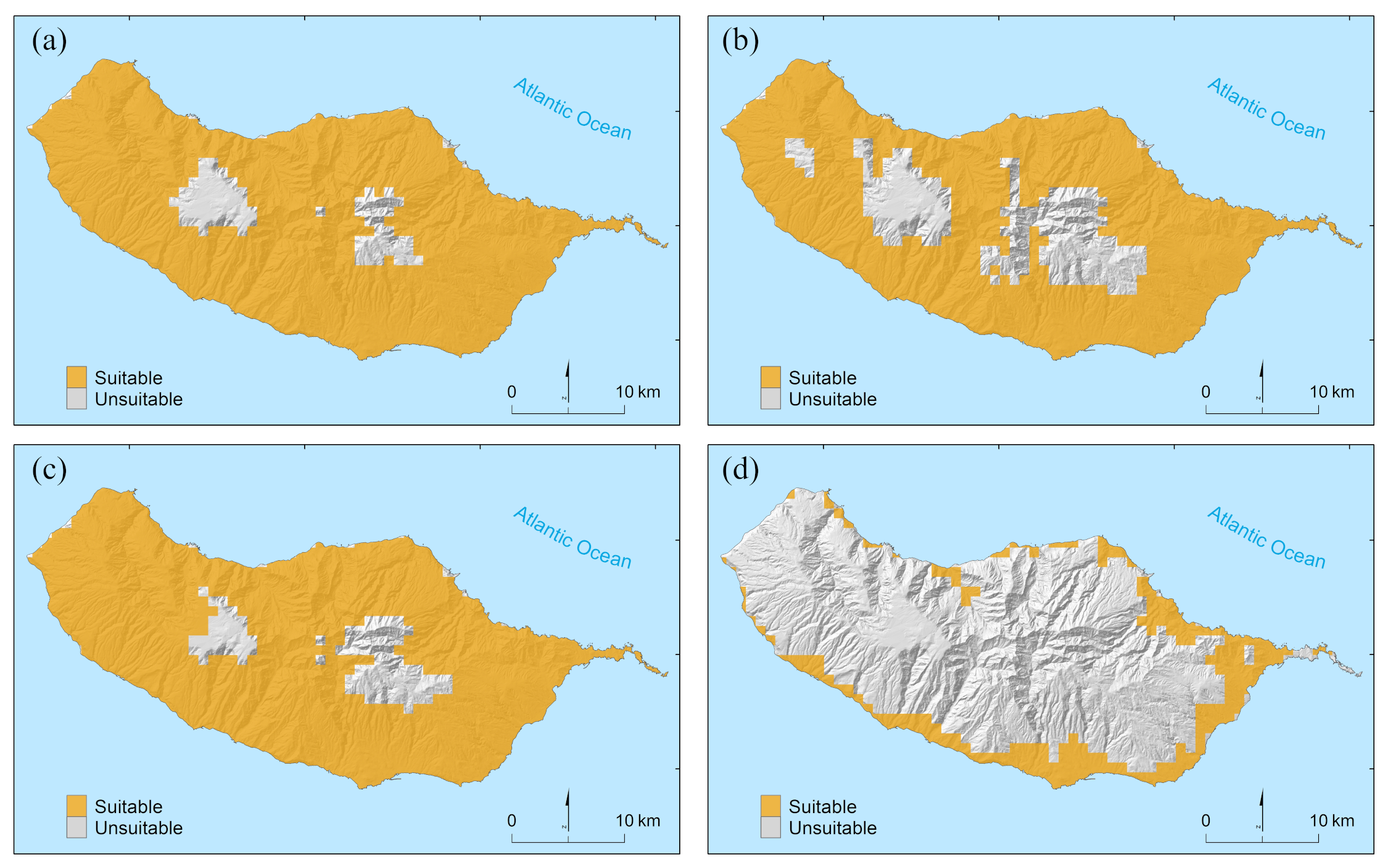

Supplement: S7 Fig — Predictions obtained for distinct modelling algorithms based on climatic conditions projected for the mid of the 21st century (2041–2060) under RCP2.6 BRT(a), GAM (b), GLM (c) an RF (d) (source of shapefile: https://www.dgterritorio.gov.pt/cartografia/cartografia-tematica/caop). (TIF) [file pntd.0010715.s008.tif]

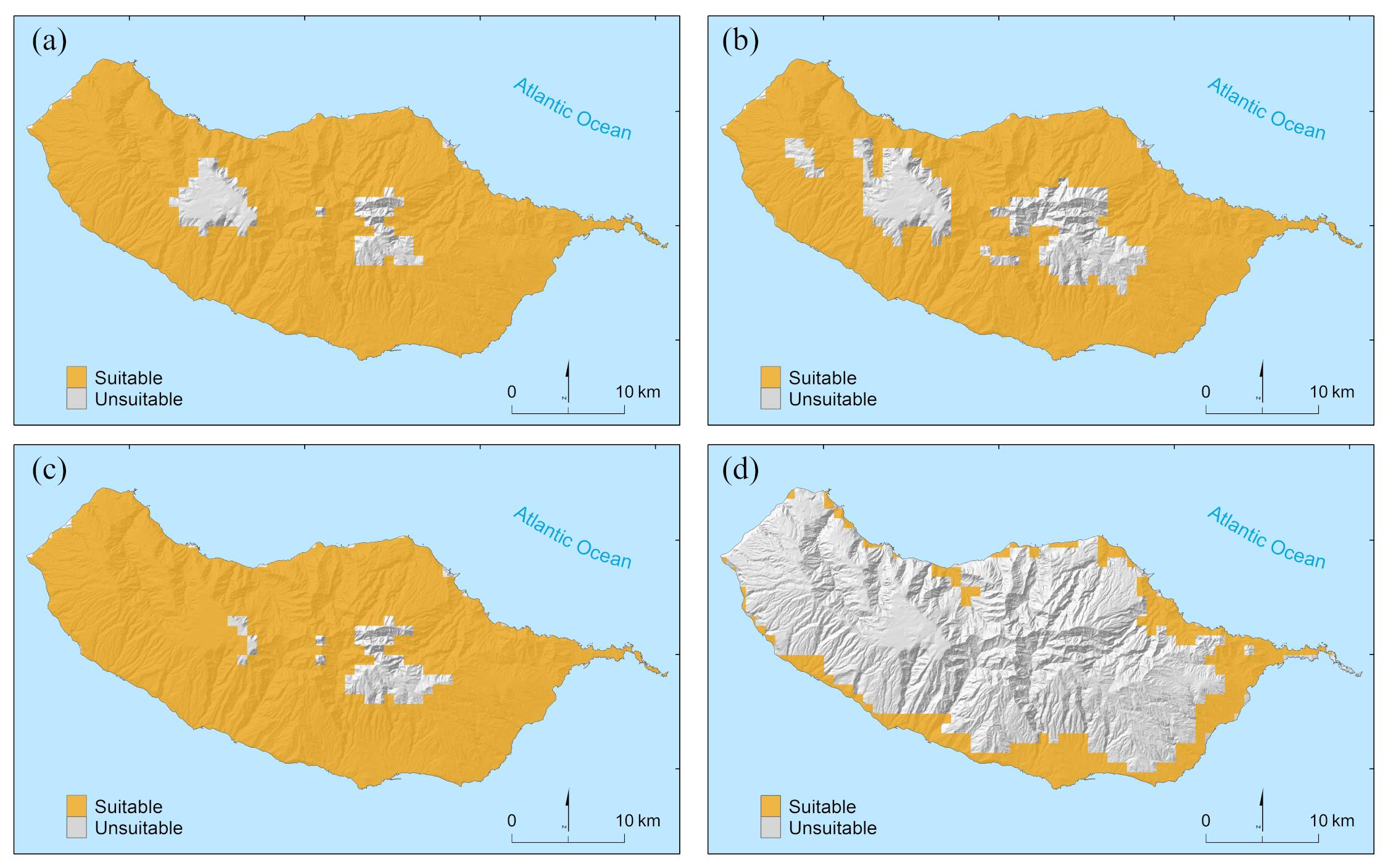

Supplement: S8 Fig — Predictions obtained for the distinct modelling algorithms based on climatic conditions projected for the mid of the 21st century (2041–2060) under RCP4.5 BRT(a), GAM (b), GLM (c) an RF (d) (source of shapefile: https://www.dgterritorio.gov.pt/cartografia/cartografia-tematica/caop). (TIF) [file pntd.0010715.s009.tif]

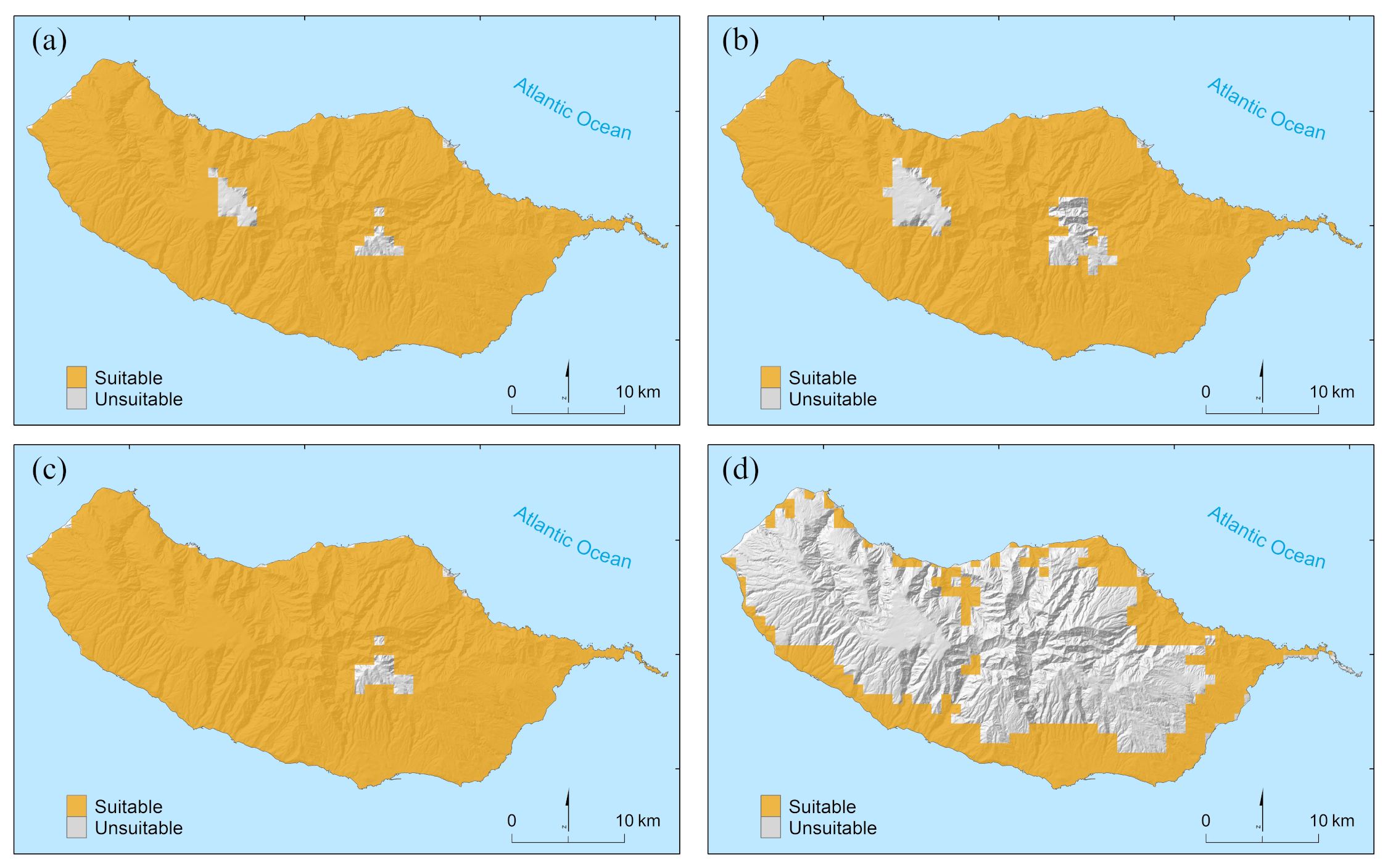

Supplement: S9 Fig — Predictions obtained for the distinct modelling algorithms based on climatic conditions projected for the mid of the 21st century (2041–2060) under RCP8.5 BRT(a), GAM (b), GLM (c) an RF (d) (source of shapefile: https://www.dgterritorio.gov.pt/cartografia/cartografia-tematica/caop). (TIF) [file pntd.0010715.s010.tif]

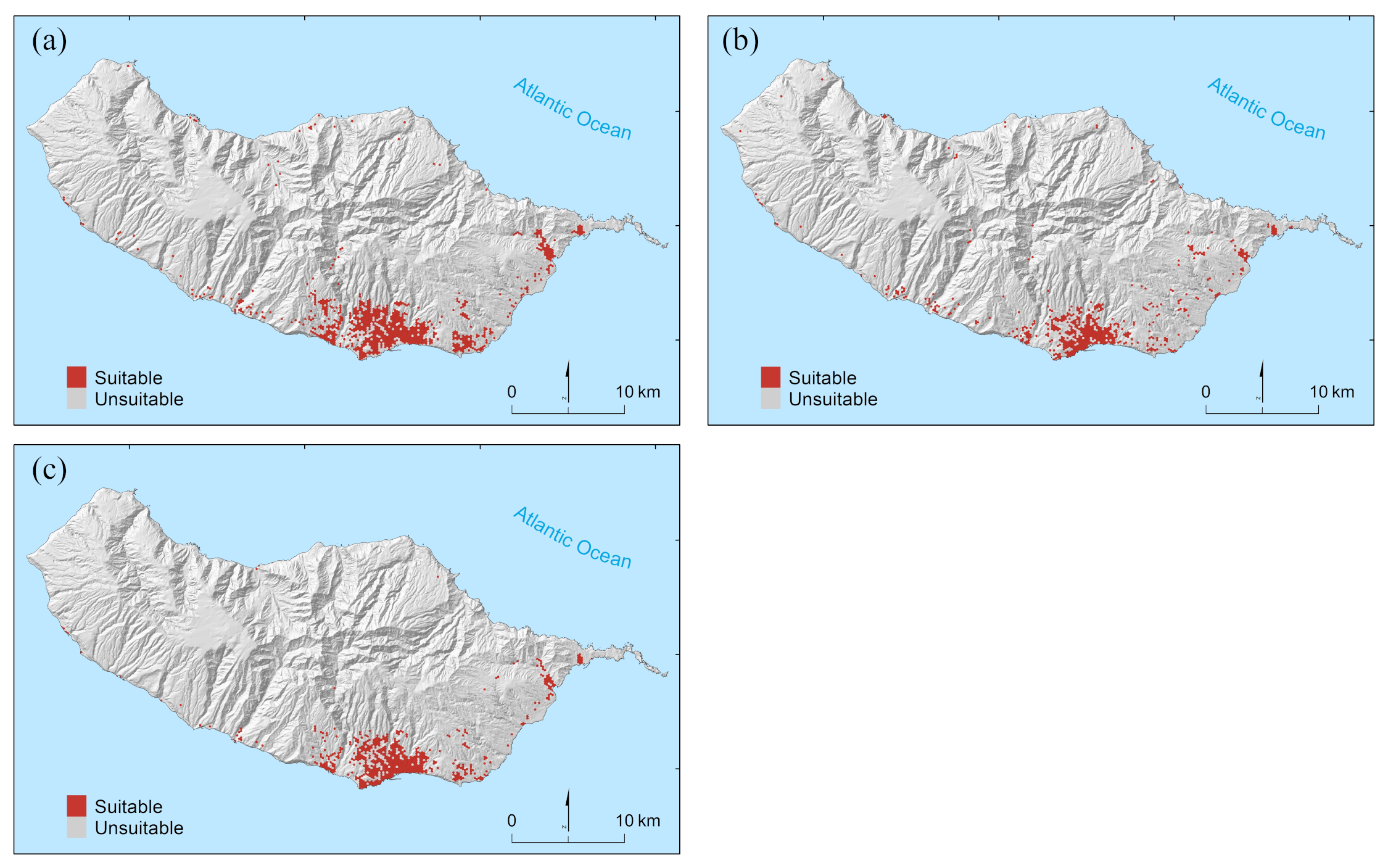

Supplement: S10 Fig — (TIF) [file pntd.0010715.s011.tif]
